# DNA methylation analysis of SFRP2, GATA4/5, NDRG4 and VIM for the detection of colorectal cancer in fecal DNA

**DOI:** 10.3892/ol.2014.2413

**Published:** 2014-08-04

**Authors:** HONGNA LU, SHILIANG HUANG, XIE ZHANG, DANPING WANG, XUESONG ZHANG, XIAOGANG YUAN, QIUBO ZHANG, ZHIGANG HUANG

**Affiliations:** 1 Ningbo Medical Center, Li Huili Hospital, Ningbo, Zhejiang 315040, P.R. China; 2 Medical School of Ningbo University, Ningbo, Zhejiang 315211, P.R. China

**Keywords:** colorectal cancer, methylated DNA, fecal DNA

## Abstract

Aberrantly methylated genes are increasingly being established as biomarkers for the detection of colorectal cancer (CRC). In the present study, the methylation levels of the secreted frizzled-related protein gene 2 (*SFRP2)*, GATA binding protein 4/5 (*GATA4*/*5*), N-Myc downstream-regulated gene 4 (*NDRG4*) and vimentin (*VIM*) promoters were evaluated for their use as markers in the noninvasive detection of CRC. Methylation-specific polymerase chain reaction was performed to analyze promoter CpG methylation of *SFRP2*, *GATA4*/*5*, *NDRG4* and *VIM* in the fecal DNA of 56 patients with CRC and 40 individuals exhibiting normal colonoscopy results. Promoter methylation levels of *SFRP2*, *GATA4*/*5*, *NDRG4* and *VIM* in CRC patients were 57.1% (32/56), 42.9% (24/56), 83.9% (47/56), 28.6% (16/56) and 41.1% (23/56), respectively. Furthermore, the specificity of the genes were 90.0% (4/40), 95.0% (2/40), 82.5% (7/40), 97.5% (4/40) and 85.0% (6/40), respectively. The overall sensitivity of detection for fecal DNA with at least one methylated gene was 96.4% (54/56) in CRC patients. By contrast, only 14 of the 40 normal individuals exhibited methylated DNA in the aforementioned promoter regions. Methylation of the *SFRP2*, *GATA4*/*5*, *NDRG4* and *VIM* promoters in fecal DNA is associated with the presence of colorectal tumors. Therefore, the detection of aberrantly methylated DNA in fecal samples may present a promising, noninvasive screening method for CRC.

## Introduction

Colorectal cancer (CRC) is the third most common type of cancer and one of the leading causes of cancer-associated mortality worldwide. Due to the long asymptomatic preclinical phase, the early diagnosis and treatment of CRC is critical for reducing disease-associated morbidity and mortality (1). The commonly used CRC screening methods include fecal occult blood testing (FOBT), barium enema, flexible sigmoidoscopy and colonoscopy. Among these, FOBT and colonoscopy are used most frequently. FOBT is relatively simple, however, it exhibits low sensitivity and specificity. Colonoscopy is considered the ‘gold standard’ for CRC screening, although, its invasive nature and the complex preparation required (colonic lavage), leave numerous patients reluctant to undergo the procedure (2). It is therefore imperative to develop an accurate and noninvasive screening test for the early detection of CRC.

CRC is the consequence of the accumulation of genetic and epigenetic modifications in colonic mucosal cells, culminating in the transformation of a benign neoplasm into a malignant tumor. Epigenetic alterations are heritable changes in gene activity and expression that occur without any alterations in the DNA sequence (3). Aberrant methylation of CpG islands in gene promoter regions are commonly associated with gene silencing and have been found to be crucial in CRC progression. Aberrant methylation often occurs during the early phases of CRC carcinogenesis (4). An increasing number of hypermethylated genes have been reported to be suitable for use as biomarkers in the detection of CRC in fecal DNA, indicating that fecal DNA methylation analysis may present a promising, noninvasive approach for the screening of early CRC (5–9). Previous studies investigating the detection of a combination of O(6)-methylguanine-DNA methyltransferase, human Mut L homolog 1 and vimentin (*VIM*) gene methylation in fecal DNA from patients with CRC, adenoma and normal individuals, reported a sensitivity of 75% in CRC and 60% in adenoma patients, and a specificity of 86%, respectively (5). These studies indicated that hypermethylated gene panels may improve the sensitivity of noninvasive screening of CRC.

In the present study, to evaluate the feasibility of fecal DNA methylation as a noninvasive CRC screening method, the methylation status of five gene promoters was investigated in fecal DNA from CRC and normal controls. The genes evaluated were secreted frizzled-related protein gene 2 (*SFRP2)*, GATA binding protein 4/5 (*GATA4*/*5*), N-Myc downstream-regulated gene 4 (*NDRG4*) and *VIM*.

## Materials and methods

### Patients and stool samples

Stool samples were obtained from 56 patients with CRC undergoing surgery or colonoscopy and 40 endoscopically normal individuals undergoing colonoscopy at Li Huili Hospital (Ningbo, China) between July 2011 and May 2012. The mean age of patients in the cancer and control groups was 60.60±12.19 years and 59.80±12.10 years, respectively. The ratio of male to female patients in the cancer group was 21:35, and that of the controls was 24:16. All subjects provided written informed consent for their participation prior to enrollment in this study and ethical approval was obtained from the ethics committee of Ningbo Medical Center, Li Huili Hospital. Stool samples were collected from all normal subjects and 40 CRC patients during the week prior to colonic lavage for colonoscopy or surgery. The remaining 16 CRC stool samples were collected 7–14 days following the initial colonoscopy. Samples were sent to the laboratory within 1 h of defecation and stored at −80°C.

### Isolation of DNA

DNA was isolated from frozen stool samples (180–220 mg) using the QiaAmp DNA Stool mini-kit (Qiagen, Hilden, Germany), according to the manufacturer’s instructions. The purified DNA was stored at −20°C for use in the experiments.

### Sodium bisulfite conversion

The methylation status of a DNA sequence may be determined using sodium bisulfite, whereby bisulfite converts unmethylated cytosine residues to uracil, leaving the methylated cytosines unchanged (10). Sodium bisulfite conversion and DNA recovery were performed using the EpiTect Bisulfite Kit (Qiagen) according to the manufacturer’s instructions, using a total of 2 μg DNA obtained from the stools. Briefly, the DNA was diluted in 40 μl RNase-free water, to which 85 μl Bisulfite Mix and 15 μl DNA Protect Buffer was added, in 200-μl in polymerase chain reaction (PCR) tubes. The following cycles were performed to convert the DNA: The DNA was denatured for 5 min at 95°C and incubated for 25 min at 60°C, then denatured again at 95°C for 5 min and incubated at 60°C for 85 min, followed by 95°C for 5 min, 60°C for 175 min, and then held at 20°C indefinitely. Following bisulfite treatment, DNA was ethanol-precipitated, resuspended in 39 μl elution buffer and stored at −20°C.

### Methylation-specific (MSP) PCR

The methylation of the *SFRP2*, *GATA4*/*5*, *NDRG4* and *VIM* promoters in the bisulfite-modified DNA was investigated using MSP PCR and primer pairs designed to discriminate between methylated and unmethylated alleles. The nucleotide sequences of the primers are shown in Table I.

Each 50 μl PCR mix consisted of 2 μl of bisulfite-modified DNA, 1X KAPA2G buffer (10 μl; Kapa Biosystems, Inc., Wilmington, MA, USA), 0.2 mmol/l deoxynucleotide triphosphate mix (1 μl; Kapa Biosystems), 0.5 μmol/l of each primer (1 μl) and 0.5 units of KAPA2G^TM^ Robust Hotstart DNA Polymerase (Kapa Biosystems). The thermocycler conditions were as follows: 95°C for 5 min, 10 cycles of 95°C for 30 sec, T_m_ (−0.8°C) for 30 sec, 72°C for 60 sec, and then 38 cycles of 95°C for 30 sec, T_m_ (°C) for 30 sec, and 72°C for 60 sec, followed by a final extension step for 10 min at 72°C. PCR products were electrophoresed on a 2.5% agarose gel and visualized under UV light. Each sample was subjected to MSP for all genes, and all MSP assays were performed in triplicate to validate the results. Those who performed the assays were blinded to all clinical information.

### Statistical analysis

The sensitivity and specificity [with 95% confidence interval (CI)] of the fecal DNA assays were calculated. To compare the characteristics of the different groups of patients, χ^2^ tests and Fisher’s exact tests were used. Odds ratios (OR) and the corresponding 95% CIs were used to assess the association between DNA hypermethylation of *SFRP2*, *GATA4*/*5*, *NDRG4* and *VIM*. All statistical tests were performed using SPSS version 11.0 software (SPSS, Inc., Chicago, IL, USA). All values were two-sided and P<0.05 was considered to indicate a statistically significant difference.

## Results

**Figure 1:**
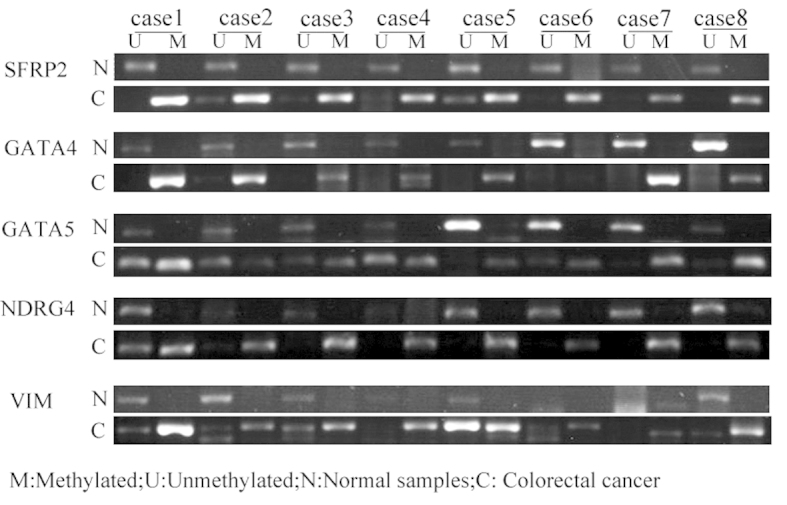
Methylation of SFRP2, GATA4/5, NDRG4 and VIM in fecal DNA obtained from colorectal cancer patients and controls, as shown by methylation-specific polymerase chain reaction using primers for methylated and unmethylated alleles of bisulfite-modified DNA. SFRP2, secreted frizzled-related protein 2; GATA4/5, GATA binding protein 4/5; VIM, vimentin; NDRG4, N-Myc downstream-regulated gene 4; M, methylated; U, unmethylated; N, normal samples; C, colorectal cancer samples.

Stool samples were collected from 56 patients with CRC (mean age, 59.8 years), and 40 endoscopically diagnosed healthy controls (mean age, 60.6 years), MSP was performed on all 96 samples and each sample was subjected to MSP for all genes (Fig. 1).

**Figure 2:**
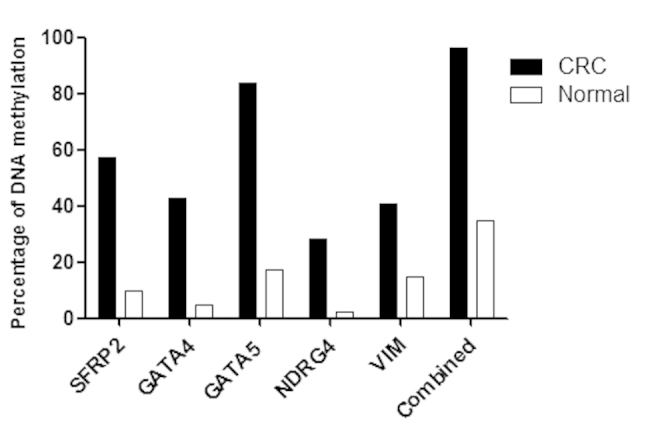
Prevalence of hypermethylated SFRP2, GATA4/5, NDRG4 and VIM in stool samples from CRC patients and normal controls. CRC, colorectal cancer; SFRP2, secreted frizzled-related protein 2; GATA4/5, GATA binding protein 4/5; NDRG4, N-Myc downstream-regulated gene 4; VIM, vimentin.

In CRC patients, *SFRP2*, *GATA4*/*5*, *NDRG4* and *VIM* were found to be methylated at levels of 57.1% (95% CI, 44.14–69.23%), 42.9% (95% CI, 30.77–55.86%), 83.9% (95%CI, 72.19–91.31%), 28.6% (95% CI, 18.42–41.48%) and 41.1% (95% CI, 29.17–54.12%), respectively. The specificity of these genes was found to be 90.0% (95% CI, 76.95–96.04%), 95.0% (95% CI, 83.5–98.62%), 82.5% (95% CI, 68.05–91.25%), 97.5% (95% CI, 87.12–99.56%) and 85.0% (95% CI, 70.93–92.94%), respectively. The overall sensitivity of the detection of fecal DNA exhibiting at least one methylated gene was 96.4% (95% CI, 87.88–99.02%) in CRC patients. By contrast, only 14 of the 40 normal individuals exhibited methylated DNA, with a specificity of 65% (95% CI, 49.51–77.87%) (Fig. 2).

The ORs for predicting the presence of CRC using the methylation of *SFRP2*, *GATA4*, *GATA5*, *NDRG4* and *VIM* were 12.00 (95% CI, 3.76–38.30; P<0.01), 14.25 (95% CI, 3.13–64.97;P<0.01), 24.62 (95% CI, 8.33–72.74; P<0.01), 15.60 (95% CI, 1.97–123.36; P=0.001) and 3.95 (95% CI, 1.43–10.93; P=0.006), respectively. With the combined analysis of the five methylation markers, the odds ratio was 50.14 (95% CI, 10.60–237.12; P<0.01)(Table II).

To compare the characteristics of the different groups of patients, χ^2^ and Fisher’s exact tests were used. The associations between clinicopathological characteristics of the CRC patients and the methylation statuses of *SFRP2*, *GATA4*, *GATA5*, *NDRG4* and *VIM* are shown in Table III. No statistically significant differences were identified with respect to patient gender, age, tumor-node-metastasis (TNM) stage or tumor location.

## Discussion

Although screening for CRC in individuals aged >50 years has been shown to reduce the incidence and mortality of CRC, numerous patients do not undergo colonoscopy-based screening due to its invasive nature (11,12). FOBT, which is a noninvasive form of CRC screening, is currently widely used. However, the sensitivity of FOBT is only 15–35%, and a substantial proportion of tumors that are not associated with bleeding remain undetected (13). Since colon cancer cells are continuously shed into the colonic lumen and released into the stool, including cells from early-stage cancer growths, molecular tests for genetic and epigenetic alterations in fecal DNA have been proposed as feasible screening methods for the early detection of colorectal neoplasias (14). Previous studies have demonstrated the potential feasibility of detecting DNA mutations in the feces of CRC patients. Ahlquist *et al* (15) analyzed freezer-archived stools from 22 patients with CRC, 11 of which with adenomas of >1 cm, and 28 normal subjects. The assay targets included point mutations at any of the 15 commonly mutated sites on the *K-ras*, *p53* and *APC* genes. Sensitivity was 91% for cancer and 82% for adenomas, with a specificity of 93%. However, due to the high cost of the multitarget panels and a difficult collection process, the clinical application of CRC screening using DNA mutation detection has been limited. Detecting epigenetic alterations in fecal DNA has increasingly been considered as an effective approach for the detection of colorectal neoplasias (16). Currently, an increasing number of hypermethylated genes in stool samples have been reported as potential biomarkers for the detection of colorectal neoplasias. Using methylated genes in the feces of CRC patients, a meta-analysis demonstrated an overall sensitivity of 62% and a specificity of 80% for colorectal neoplasia (17).

In the present study, the feasibility of detecting methylated DNA in stool samples was evaluated as a noninvasive screening tool. The evaluation of five methylation markers, *SFRP2*, *GATA4*/*5*, *NDRG4* and *VIM*, revealed that >96% of patients with CRC and only 35% of normal controls exhibited at least one methylated allele in their fecal samples. Among the five genes, two demonstrated >50% sensitivity in fecal DNA from CRC patients and all five genes exhibited >60% specificity in controls. The *GATA5* methylation marker demonstrated the highest sensitivity (83.9%) in stool samples from individuals with CRC. No association was identified between the presence of methylated fecal DNA and patient gender, age, TNM stage or tumor location. Overall, these results indicated a correlation between early-stage and later-stage CRC, and that this method of detection may have equivalent sensitivity in proximal and distal cancers. Thus, the analysis of fecal DNA methylation may present a useful and noninvasive method of screening for colorectal neoplasia.

All five methylation markers of the genes selected for this study have been identified in CRC previously. The *SFRP2* gene belongs to a recently established category of tumor suppressor genes, *SFRPs*, and silencing of *SFRPs* via promoter methylation causes constitutive activation of the Wnt/β-catenin signaling pathway, which is associated with multiple tumors, including CRC (18). Müller *et al (*19) reported that *SFRP2* hypermethylation exhibits a sensitivity of 77–90% with regard to identifying patients with CRC. Huang *et al* (20) reported that methylation of SFRP2 occurs in 94.2% of patients with CRC, with occurrences of 52.4, 37.5 and 16.7% in adenomas, hyperplastic polyps and ulcerative colitis, respectively. In this study, methylated *SFRP2* was detected in the stool samples of Chinese patients with CRC and normal individuals, indicating that it is an effective marker suitable for detecting CRC, with a sensitivity of 57.1% and specificity of 70%. However, these values were lower than those reported in previous studies (18,19).

The methylation of *GATA4* is also a frequent and specific event in CRC. *GATA4* is a regulatory transcription factor that suppresses upstream Dab2. Methylation of the CpG islands of the promoters of this tumor suppressor gene may lead to gene silencing. Hellebrekers *et al* (21) investigated *GATA4* methylation in the fecal DNA of CRC patients and controls, and found it to exhibit a sensitivity of 59% and specificity of 88% for CRC detection. The results of the present study identified methylated *GATA4* in 42.9% of CRC fecal samples.

*NDRG4* has been investigated as a possible tumor suppressor, and *NDRG4* mRNA and protein expression were found to be lower in CRC than in control samples, which was observed to correlate with the methylation status of the promoters of this gene. Melotte *et al* (22) investigated *NDRG4* promoter methylation as a biomarker for the early detection of CRC in fecal samples, whereby it demonstrated a sensitivity of 56% and a specificity of 96%. However, the present study revealed a lower sensitivity (28.57%) in CRC detection.

In the present study, the three aforementioned methylated genes were used to investigate the utility of fecal DNA in CRC detection. The results revealed that the specificity and sensitivity of SFRP2 (90.0 and 57.1%, respectively), GATA4 (95 and 42.9%, respectively) and NDRG4 (97.5 and 28.57%) were lower than those reported in previous studies. These differences may be due to the different methods of detecting methylation, differences in technical personnel and differences in patient populations.

The *VIM* gene, a marker of mesenchymal cells, encodes a protein constituent of intermediate filaments and has been demonstrated to be transcriptionally silent in normal colorectal epithelial crypt cells (23). Aberrant *VIM* methylation may be detected in the fecal DNA of CRC patients; however, it is rarely identified in normal subjects. Furthermore, using the *VIM* gene methylation marker as a noninvasive method for early CRC has been commercialized (23). Chen *et al* (24) observed 46% sensitivity with 90% specificity for CRC detection in the aberrant *VIM* exon 1 and methylation of fecal DNA, indicating hypermethylation. In the present study, results associated with *VIM* (methlyation levels of 41.1%)were comparable to those of previous studies.

Among the five genes investigated in this study, *GATA5* demonstrated the most potential as a methylation marker for CRC screening, as it exhibited the highest sensitivity (83.9%) for CRC in individual fecal samples. To evaluate the potential of *GATA5* as a methylation marker for CRC detection, Hellebrekers *et al* (21) analyzed large groups of CRC patients and controls, and observed a high frequency of *GATA5* methylation in CRC (79%) and low levels in normal colorectal mucosa (13%). In the present study, the methylation of *GATA5* in fecal DNA for CRC detection exhibited a higher sensitivity (83.9%) than in the study by Hellebrekers *et al* (79%) (21). Although the sensitivities were different, the two studies indicated that the methylation of *GATA5* in fecal DNA may present a potential biomarker for colorectal tumors.

In conclusion, the results of the present study demonstrated the feasibility of using multiple methylation markers as a noninvasive method for detecting early CRC. Further studies are required to refine the panel of potential methylation markers for CRC.

## Figures and Tables

**Table I:** Summary of the primer sequences, PCR product size obtained and annealing temperature used for MSP assays.

Gene	Primer	Sequence (5′-3′)	Annealing temperature (°C)	PCR product size (bp)
SFRP2	M	F: 5′-TTTTTGTAGGGGCGTTTTTATAAC-3′	54	163
		R: 5′-TATCGATATACTCCCCAATACCG-3′		
	U	F: 5′-AGATTTTTGTAGGGGTGTTTTTATAAT-3′	52	163
		R: 5′-ACCTATCAATATACTCCCCAATACCA-3′		
GATA4	M	F: 5′-GTCGGGATAGTTTTTCGTTC-3′	52	134
		R: 5′-CGATTTAAAACCGACAATCA-3′		
	U	F: 5′-AAGGTTGGGATAGTTTTTTGTTT-3′	50	134
		R: 5′-TCCCAATTTAAAACCAACAATCA-3′		
GATA5	M	F: 5′-TTAGAAATCGAGGAAATCGC-3′	54	133
		R: 5′-GTAAACCCCCTCGTTACGTA-3′		
	U	F: 5′-TGTTTAGAAATTGAGGAAATTGT-3′	48	133
		R: 5′-CCCATAAACCCCCTCATTACATA-3′		
NDRG4	M	F: 5′-GGAGTTTAAATAAAGATTACGGTAGC-3′	50	103
		R: 5′-ATACGCTACGAAACCCTACC-3′		
	U	F: 5′-GGGAGTTTAAATAAAGATTATGGTAGT-3′	48	103
		R: 5′-AATACACTACAAAACCCTACC-3′		
VIM	M	F: 5′-AGGAAAGTATAAATTTCGGGTGC-3′	52	173
		R: 5′-ATAAACGACGTCTTTCACCCTTAC-3′		
	U	F: 5′-AAAAGGAAAGTATAAATTTTGGGTGT-3′	48	173
		R: 5′-TATAAACAACATCTTTCACCCTTACCT-3′		

PCR, polymerase chain reaction; MSP, methylation-specific; bp, base pair; SFRP2, secreted frizzled-related protein 2; GATA4/5, GATA binding protein 4/5; NDRG4, N-Myc downstream-regulated gene 4; VIM, vimentin; M, methylated; U, unmethylated; F, forward; R, reverse.

**Table II:** Comparison of predictive power between SFRP2, GATA4/5, NDRG4, Vimentin and combined for colorectal cancer.

Gene	Sensitivity, % (95% CI)	Specificity, % (95% CI)	Odds ratio (95% CI)	P-value
SFRP2	57.14 (44.14–69.23)	90.0 (76.95–96.04^a^)	12.00 (3.76–38.30)	2.55×10^−6^
GATA4	42.86 (30.77–55.86)	95.0 (83.5–98.62^a^)	14.25 (3.13–64.97)	3.9×10^−5^
GATA5	83.93 (72.19–91.31)	82.5 (68.05–91.25^a^)	24.60 (28.33–72.74)	9.91×10^−4^
NDRG4	28.57 (18.42–41.48)	97.5 (87.12–99.56^a^)	15.60 (1.97–123.36)	0.001
VIM	41.07 (29.17–54.12)	85.0 (70.93–92.94)	3.95 (1.43–10.93)	0.006
Combined	96.43 (87.88–99.02^a^)	65.0 (49.51–77.87^a^)	50.14 (10.60–237.12)	6.65×10^−11^

a P = 1, no difference was identified between the two groups.

SFRP2, secreted frizzled-related protein 2; GATA4/5, GATA binding protein 4/5; NDRG4, N-Myc downstream-regulated gene 4; VIM, vimentin; CI, confidence interval.

**Table III:** Association between DNA hypermethylation and clinicopathological characteristics of colorectal cancer.

Characteristics	Cases, n	SFRP2	GATA4	GATA5	NDRG4	VIM	Combined
Colorectal cancer	56	32/56 (57.1)	24/56 (42.9)	47/56 (83.9)	16/56 (28.6)	23/56 (41.1)	54/56 (96.4)
Gender							
Male	21	10/21 (47.6)	12/21 (57.1)	17/21 (81.0)	6/21 (28.6)	7/21 (33.3)	20/21 (95.2)
Female	35	22/35 (62.9)	12/35 (34.3)	30/35 (85.7)	10/35 (28.6)	16/35 (45.7)	34/35 (97.1)
P-value		0.265	0.094	0.925	1	0.362	1
Age, years							
≤50	10	6/10 (60.0)	4/10 (40.0)	9/10 (90.0)	2/10 (20.0)	5/10 (50.0)	10/10 (1.0)
>50	46	26/46 (56.5)	20/46 (43.5)	38/46 (82.6)	14/46 (30.4)	18/46 (39.1)	44/46 (95.7)
P-value		1	1	0.919	0.783	0.781	1
TNM stage							
I/II	32	16/32 (50.0)	12/32 (37.5)	25/32 (78.1)	7/32 (21.9)	13/32 (40.6)	30/32 (93.8)
III/IV	24	16/24 (66.7)	12/24 (50.0)	22/24 (91.7)	9/24 (37.5)	10/24 (41.7)	24/24 (1.0)
P-value		0.212	0.35	0.318	0.2	0.938	0.5
Location							
Rectum	38	23/38 (60.5)	13/38 (34.2)	29/38 (76.3)	10/38 (26.3)	16/38 (42.1)	36/38 (94.7)
Right colon	6	3/6 (50.0)	3/6 (50.0)	6/6 (1.0)	2/6 (33.3)	2/6 (33.3)	6/6 (1.0)
Left colon	12	6/12 (50.0)	8/12 (66.7)	12/12 (1.0)	4/12 (33.3)	5/12 (41.7)	12/12 (1.0)
P-value		0.768	0.131	0.079	0.865	0.92	0.6123
Normal control	40	4/40 (10.0)	2/40 (5.0)	7/40 (17.5)	1/40 (2.5)	6/40 (15.0)	14/40 (35.0)

SFRP2, secreted frizzled-related protein 2; GATA4/5, GATA binding protein 4/5 NDRG4, N-Myc downstream-regulated gene 4; VIM, vimentin; TNM, tumor node metastasis.
